# Extinction Debt and Colonizer Credit on a Habitat Perturbed Fishing Bank

**DOI:** 10.1371/journal.pone.0166409

**Published:** 2016-11-28

**Authors:** Daniel E. Duplisea, Michael G. Frisk, Verena M. Trenkel

**Affiliations:** 1 Fisheries and Oceans Canada Canada, Institut Maurice-Lamontagne, Mont-Joli, QC, Canada; 2 School of Marine and Atmospheric Sciences, Stony Brook University, Stony Brook, NY, United States of America; 3 Ifremer, Nantes, France; Hellenic Centre for Marine Research, GREECE

## Abstract

Temporal changes in occupancy of the Georges Bank (NE USA) fish and invertebrate community were examined and interpreted in the context of systems ecological theory of extinction debt (EDT). EDT posits that in a closed system with a mix of competitor and colonizer species and experiencing habitat fragmentation and loss, the competitor species will show a gradual decline in fitness (occupancy) eventually leading to their extinction (extirpation) over multiple generations. A corollary of this is a colonizer credit, where colonizer species occupancy may increase with fragmentation because the disturbance gives that life history a transient relative competitive advantage. We found that competitor species occupancy decreased in time concomitant with an increase in occupancy of colonizer species and this may be related to habitat fragmentation or loss owing to industrialized bottom trawl fishing. Mean species richness increased over time which suggests less specialization (decreased dominance) of the assemblage that may result from habitat homogenization. These analyses also showed that when abundance of species was decreased by fishing but eventually returned to previous levels, on average it had a lower occupancy than earlier in the series which could increase their vulnerability to depletion by fishing. Changing occupancy and diversity patterns of the community over time is consistent with EDT which can be exacerbated by direct impacts of fishery removals as well as climate change impacts on the fish community assemblage.

## Introduction

Theoretical and empirical research has linked trends in species occupancy, abundance and population extinction with habitat alteration and community structure in a wide range of animal taxa. Specifically, over time, habitat fragmentation can result in increased abundance of rapidly dispersing species and the local extinction of poorer colonizers [1,2]. The extinction debt hypothesis or theory (EDT) [3] introduced the counter-intuitive idea that with continued permanent habitat destruction an ordered extinction starts with species that are the best competitors ending with the worst competitors but are the best colonizers [2,4]. Tilman's model introduced a strong tradeoff between competitive and colonizing ability; thus, when habitat is fragmented and destroyed, the best competitors do not have the dispersive ability to colonize fragmented landscapes. Theory also suggests that under equilibrium conditions, species with good competitive abilities are also the species which are likely to dominate biomass and in some ways typify fish community assemblages. At the same time, the poorer competitors, but better dispersers or colonizers, would increase in relative abundance and spatial occupancy. For example, if a community experiences habitat fragmentation and loss, an extinction debt is realized that will result in the species with the greatest competitive ability and weakest colonizing ability to trend toward local extinction (extirpation) over multiple generations. With permanent habitat destruction, extirpation becomes inevitable once the amount of suitable habitat falls below the extinction threshold. The speed at which the debt is repaid (i.e. rate that the species abundance and occupancy disappears = amortization of the debt) is proportional to the distance from threshold [4]. For populations close to but still below the threshold, the amortization period of the debt may be very long and not necessarily noticeable in the short term especially given the large natural variability of many animal populations. Additionally, for regions where suitable patches remain even with high connectivity, a debt may still be incurred, but it will be less noticeable in the shorter term and low abundance populations are more vulnerable to stochastic events [5]. In situations where habitat can regenerate at time scales close to the the generation time of the species involved, then the extinction rate is variable and the extinction threshold may not be a one way barrier. When the habitat can regenerate faster than the generation time of the species, extinction is not inevitable and in fact unlikely, but full recovery of the population may take longer than expected based on a simple demographic analysis.

In the marine environment habitat fragmentation and loss caused by bottom trawling and dredging, can reduce benthic biodiversity, community composition and biomass of megafauna and epifauna [6–11]. Trawling impacts on benthic communities is complex and related to the degree and frequency of natural disturbance and resilience of the community [12]. Sandy habitats may naturally experience a high frequency of disturbance and impacts from trawl fishing is comparatively low [12]. In contrast, pebble and rocky habitats with infrequent natural disturbance are more sensitive to the effects of fishing and are more likely to experience a reduction of species diversity following disturbance [13].

**Fig 1:**
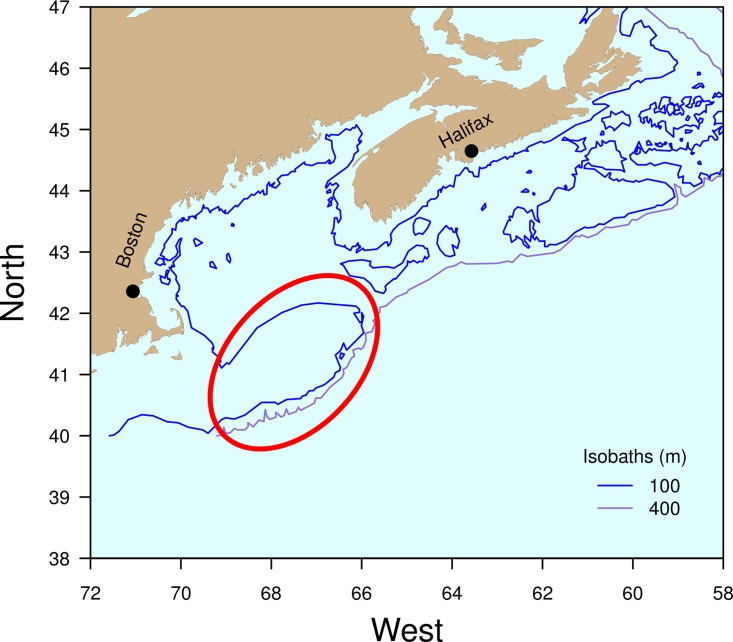
Bathymetric map of the northeastern United States and southeastern Canada showing Georges Bank circled on the map.

Georges Bank is a productive ecosystem in the northwestern Atlantic (Fig 1) that has supported prolific fisheries for over two centuries [14]. Since about 1980, the GB finfish community has gone through major changes related to fishing, large-scale species movement and climate change [14–17]. GB has a wide range of habitat types including sands, granules-pebbles, cobbles and boulders [18]. The effects of bottom trawling and dredging have been well documented showing declines in benthic productivity, species richness and increased homogenization [7,9]. However, the expected effects of habitat homogenization on mobile fish are more complex compared to the physical disturbance of sedentary benthic communities. Temporal and spatial dynamics of mobile species depend on a range of habitats critical for successive life stages.

Hanskii et al. [19], pointed out that in the course of an extinction debt there will be a “transient” period with increased prevalence of rarer species. This results because of the time-lag between fragmentation and eventual extinction and is especially important for species close to their extinction threshold. Thus, evaluating the present conditions in an ecosystem may not reflect the actual extinction debt accrued or potential future population extinctions resulting from habitat destruction. When perturbations destroy habitat it becomes fragmented and less structured and eventually homogenized, and counter-intuitively, local species richness may actually increase during the transient period [20] followed eventually by richness declines. As spatially structured habitat becomes increasingly fragmented, boundaries between heterogeneous habitat types are blurred and competitors with specialized niches are forced to occupy suboptimal habitat which reduces their fitness. The key feature that emerges is that a greater number of species will occupy a greater proportion of area leading to greater average richness per sampled area because of the habitat homogenization.

Extinction debt theory was developed for terrestrial ecosystems that are comparatively easier to survey than marine systems [21]. Frisk et al. 2011 [17], studying the GB finfish and shellfish community, and the related concept of occupancy and abundance (O-A) relationships, found that the strengths and slopes of the relationship declined between 1963–2006 and was correlated to trawling effort. The changes in O-A relationships are consistent with habitat fragmentation as has been well documented for terrestrial systems [22,23]. It is important to recognize that EDT is a theoretical model with a hard tradeoff between colonizing and competitive abilities [24]. This hard tradeoff needs caution when applied in marine environments because of the a wide range of life histories and dispersal levels displayed from passive large-scale pelagic eggs, to nesters and live bearers [25] and marked ontogenetic niche shifts that includes differences in the strength and types of habitat dependence. However, when the theoretical underpinnings of EDT are combined with empirical observations, the resulting analysis can serve as a tool to evaluate the state of the system and provide information critical for understanding trends in species richness.

In this study we consider long term changes in species richness spatial distribution (occupancy) in the Georges Bank ecosystem in the context of potential habitat homogenization and transient signs of extinction debt that could be manifested in increased species richness and changing occupancy. We specifically examined OA trends for groups of species that correspond to Competitor-Colonizer groups to address the dynamics of competitive skill and dispersal in marine systems. The overall objective is to determine if long term trends in the finfish and shellfish community are consistent with a system experiencing extinction debt from habitat alteration and factors that confound or contribute to mechanistic understanding of the trends.

## Materials and Methods

### Data

We analyzed data from the autumn National Marine Fisheries Service’s (NMFS) bottom trawl survey from 1963–2008 for Georges Bank. This is a random stratified survey and consists of an average of 63 tows each year of 30 minutes each at 7 km/h. The gear used is a Yankee 36 bottom otter trawl with 12.7 mm codend liner. For every tow, all fish and invertebrates were separated into species counted and weighed. In 2009, new tows were added to the survey as well as changes to trawl mesh size, gear type and tow speed. These changes affected species relative catchability, diversity and abundance amongst other community measures; therefore, our analyses are based on data only up to and including 2008 rather than allowing a gear based artefact to influence results. Georges Bank is a relatively small area with a fairly high tow density therefore, we dealt with the results of individual station tows rather than using stratified means and the number of tows per stratum is relatively constant from year to year and most differences occurred in two consecutive years: 1978 and 1979. Furthermore, because strata are based on depth rather than relevant habitat features, information related to spatial structure and occupancy could be lost if stratified means were used in this analysis as depth does not necessarily equate to relevant habitat in the sense that habitat is being used here.

### Species filtering

The NMFS database for Georges Bank has recorded the presence of 219 different categories of catch including “trash” or broad lumped taxonomic categories with many species. For the present analyses, it was necessary to apply appropriate filtering criteria to obtain subsets of the 219 categories that reduce the possibility that poor catchability of rarely caught species would lead to spurious conclusions.

For analyses of abundance trends, we needed to confine the species to groups that were well caught by the survey and thus relative abundance and occupancy differences could be considered real. We therefore applied a filter following [17] to the dataset where the only groups considered in the analysis were those that:

(1) were caught in more than 40 of the survey’s 46 years; (2) where more than 1000 individuals were caught over the survey time series (in standardized tows); (3) identified to the species level; (4) were not shrimp as they are not well caught by the gear and are sometimes partially destroyed during sampling. This filter resulted in a dataset which included 39 species (S1 Appendix).

For analyses of species richness, we need only to be confident in presence/absence thus the filter was relaxed to allow more rarely caught species into the analysis. Accordingly, only filtering criteria (1) and (3) above were applied. This filter resulted in a data which included 164 species (S1 Appendix).

### Competitor-Colonizer group classifications

The reduced species dataset, consisting of 39 species meeting the filtering criteria above were classified as competitors (*CM*, n = 9), colonizers (*CL*, n = 9) or mixed (*MX*, n = 21) (S1 Appendix). These qualitative designations were determined through examination of Fishbase.org as well as primary publications and DFO and NMFS species information sheets and [26].

The main characteristics subjectively used for dividing species three different groups were: maximum age, fecundity, age at maturity, larval/juvenile phase dispersal. A species with a long life span and older age at maturity would be classified in the Competitors group as would species with relatively low lifetime fecundity and species having live birth. Conversely, Colonizer group species tended to have relatively high lifetime fecundity as well as an ability to disperse widely often at juvenile stages (e.g. a long pelagic phase found in many invertebrates for example). Colonizers were often of small body size. Mixed group species were those which displayed a mix of characteristics and it was not obvious how to classify a species. In some respects the mixed group is the null group which represents a lack of designation rather than a positive group assignment. There is some overlap with the groupings of [27], though the Competitor-Colonizer groups developed here do not correspond perfectly to their groupings of periodic, opportunist and equilibrium. The ecological interpretations here are based primarily on trends in the two ends of the Competitor-Colonizer classification: Competitors and Colonizers which have a more definitive classification and therefore the results have a certain degree of robustness to uncertainty in group classification.

### Analyses

Occupancy was modelled using nested generalized additive models (GAM) models to account for (1) abundance (2) abundance and competitor colonize group (3) abundance and competitor colonize group and year (4) as 3 but with a random effect for species:

O=s(log(A)) (1)

O=s(log(A))+s(log(A)byCC) (2)

O=s(log(A))+s(log(A)byCC)+s(yearbyCC) (3)

O=s(log(A))+s(log(A)byCC)+s(yearbyCC)+s(species,random) (4)

Where O is occupancy which was calculated as the proportion of stations where a species was caught in any year. A is the mean abundance of that species in the survey for a year. CC is the competitor-colonizer group factor. *s* represents a cubic spline smooth. Because occupancy is a proportion, the GAM was fitted with quasi-binomial errors. 1732 datapoints were available for modelling the 39 species dataset. Model 1 is the most basic OA model with a single fitted smoother describing occupancy as a function of log abundance. Model 2 accounts for deviations from the Model 1 smooth within competitor-colonizer groups. Model 3, is the model 2 but accounts then for a temporal effect (year) on occupancy in groups. Model 4 is the full model (model 3) with a random effect for species. For Models 2, 3 and 4 we restricted smoothers to have similar degrees of freedom within groups and smoothing terms determined using generalized cross validation. Occupancy was logit transformed and models were fitted using the logit link function. The nested models were compared to adjacent order models with ANOVA (F-test) to determine the importance of terms for explaining occupancy.

We examined the mean species richness at stations over time in the 164 species dataset. Changes in richness can be indicative of the effect on species of homogenizing habitat.

All analyses were conducted in R and specifically using the library mgcv [28].

## Results

**Fig 2:**
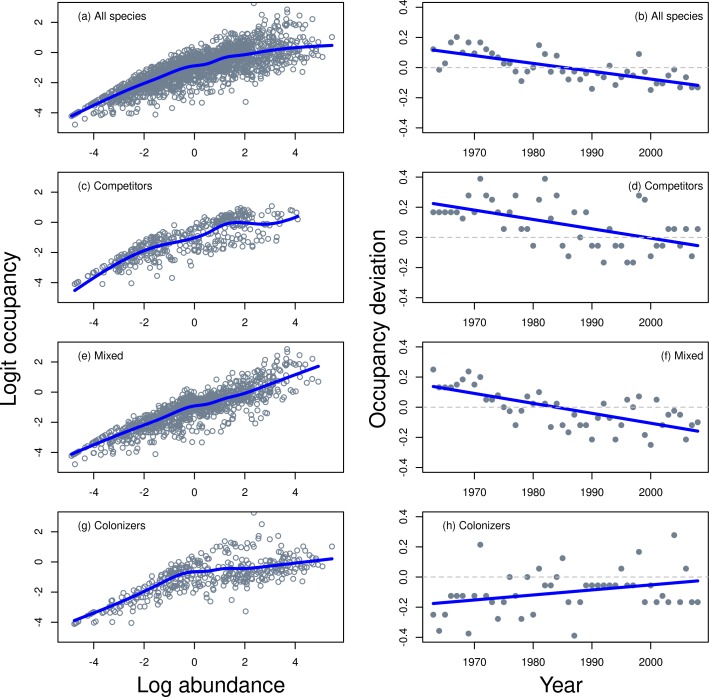
Occupancy-abundance for the 39 species Georges Bank fish community (a) and for sub groups related to Competitor-Colonizer (CC) group classifications (c,e,g). The solid line in (a,c,e,g) represents a GAM though the data with quasi-binomial errors. The occupancy deviation (OD) each year from the expected value is plotted which shows how abundance standardized occupancy deviates from the temporal mean (expected value) for all species (b) and within each CC (d,f,h).

Occupancy vs abundance of the 39 species Georges Bank dataset for all years showed the standard log asymptotic relationship for all species combined as well as just for species in different colonization groups (Fig 2 left column). The overall model (Model 1, Fig 2A) and the within CC group model (Model 2, Fig 2C, 2E and 2G) smoothing terms were significantly different than the overall group smooth (Table 1) and Model 2 was significantly better than Model 1 (ANOVA, p < 0.0001). When the data were examined in relation to their position above and below the fitted GAM line, latter years tended to fall below the smoothed line while earlier years tended to be above. Therefore, a statistic called occupancy deviation (OD) was developed to show the proportion of data points larger than predicted (above the GAM line) and smaller than predicted (below the GAM line) fit for each year (Fig 2 right column). OD represents the proportional deviation in any one year from the expected mean community value determined for all years and a trend in OD over time indicates that the OA relationship has also changed over time. OD showed a declining trend over time for all species (Fig 2B) and for Competitor (Fig 2D) and the Mixed (Fig 2F) groups. To the contrary, the OD for Colonizers (Fig 2H) increased. This indicates that for Competitors, their occupancy at the end of the time series was 10% below the mean fitted occupancy for competitor species while at the beginning of the series it was as much as 40% above the mean fitted occupancy. The GAM model analysis (Model 2, Table 1) showed that the occupancy abundance relationship was significantly different within colonizers and competitors but not for mixed species compared to the overall model.

**Table 1:** generalized additive model fits of occupancy in the Georges Bank fish community 1964–2008. Bolded values indicate a significant explanatory variable for observed occupancy of the fish and invertebrate community.

	Model 1		Model 2		Model 3		Model 4	
	F-statistic	P	F-statistic	P	F-statistic	P	F-statistic	P
**Smooth on abundance**								
**Overall**	267.5	**< 0.0001**	36.592	**< 0.0001**	0.015	0.90393	4.108	**0.000295**
**Competitors**	-	-	2.369	**0.0328**	5.53	**< 0.0001**	0	0.999771
**Mixed**	-	-	0.446	0.8184	2.675	**0.00848**	0.472	0.535501
**Colonizers**	-	-	2.832	**0.0115**	12.642	**< 0.0001**	0.036	0.968237
**Smooth on year**								
**Competitors**	-	-	-	-	7.649	**0.00081**	10.391	**0.00017**
**Mixed**	-	-	-	-	21.788	**< 0.0001**	33.419	**< 0.0001**
**Colonizers**	-	-	-	-	4.151	**0.01884**	3.571	**0.037071**
**Random effect (species)**	-	-	-	-	-	-	64.4770	**< 0.0001**
**Deviance explained**	64.6%		69.0%		70.2%		88.3%	

**Fig 3:**
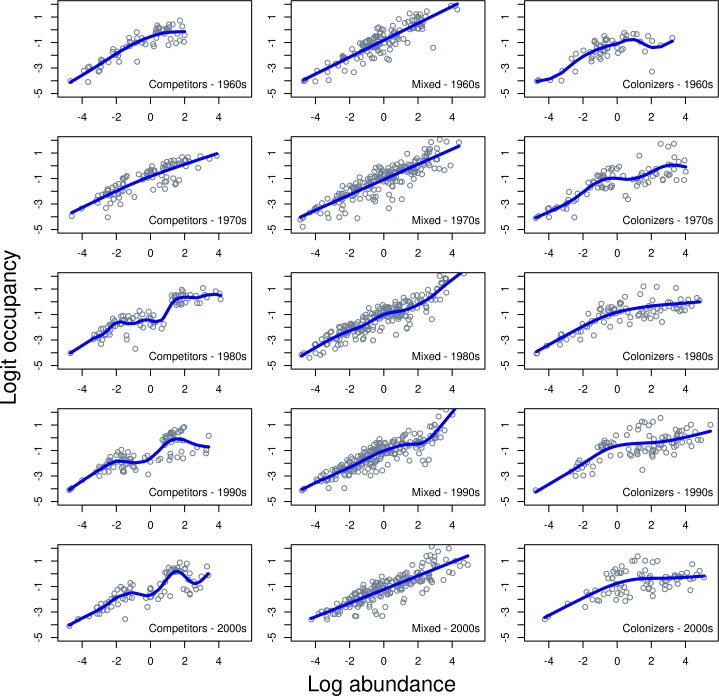
Occupancy-abundance for subgroups of the 39 species Georges Bank fish community related to Competitor-Colonizer (CC) group and a factor for Decade which the data represent. The solid lines represents a GAM through the data with quasi-binomial errors.

The temporal trend in OD suggests that the OA relationship for the whole community as well as component groups changed between 1963 and 2008 and differently between Colonizers and Competitors. Model 3 formalizes a test of a temporal trend of OA within groups (Fig 3). Model 3 is Model 2 with an additive smooth term for year within the Competitor-Colonizer groups. Model 3 was significantly better than Model 2 (ANOVA, p < 0.0001) and all the additive smoothing terms provided a significantly better fit (Table 1). This result confirms the trend shown in OD (Fig 2) where occupancy of Competitors significantly decreased over time even when that occupancy was standardized by abundance. It also indicates that the increase in occupancy in Colonizers over time was significant (Table 1). Competitor abundance increased between the 1960s and 1970s while the occupancy abundance relationship remained fairly stable, but the relationship changed more so from the 1980s onward (Fig 3). Colonizers showed increasing occupancy and abundance from the 1960s until the 2000s and a change in occupancy abundance relationship during this time.

Model 4, contains a random effect for species that explains 18% more of the variance in occupancy than Model 3 without a random effect (Table 1). Inclusion of a random effect for species still showed significant differences in occupancy over time for all the Competitor-Colonizer groups, confirming the decline in occupancy over time of competitors and increase in occupancy of colonizers. The species random effect, however, does render the differences in abundance within species groups an insignificant explanation for occupancy differences (Table 1). This suggests that changes in species assemblage over time may be an important explanation for occupancy changes on Georges Bank.

**Fig 4:**
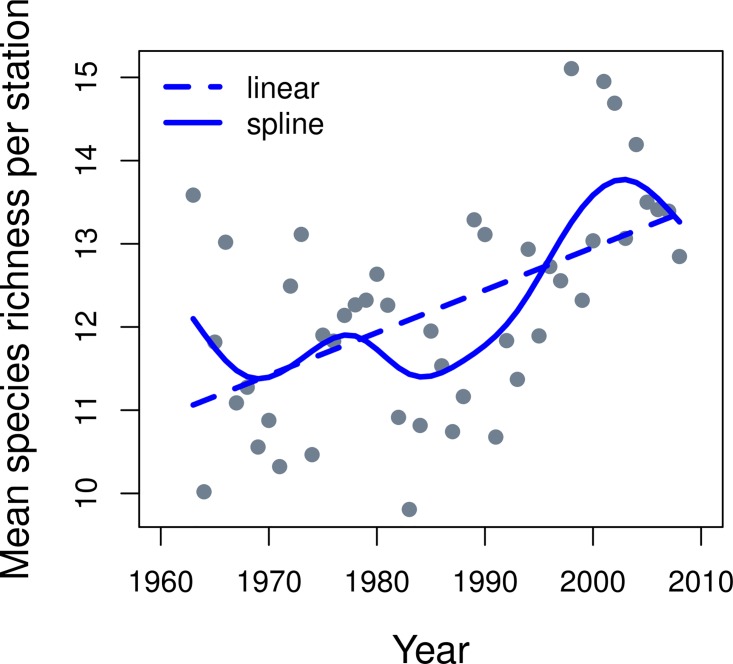
Mean species richness per station on Georges Bank from the trawl survey with the 164 species dataset. The dashed line is a significant (r2 = 0.27, p = 0.0001) straight line (least squares fit) through the data while the solid line is a smoothed curve (spline) through the data.

Mean species richness per station increased from 1963 to 2008 by about 2.5 species or about 20% (Fig 4, linear model). The smoothed GAM fitted to the same data show a more interesting curve with similar lows of about 11.4 species in 1969 and 1984 and a maximum richness of 13.8 species in 2004. The increase in richness is primarily occurring after the mid-1980s. These results were based on the 164 species dataset which specified that a species had to be caught in 40 out of 46 years in the survey to be included. Therefore this analysis does not represent an increasing occurrence of incidental catches of unfamiliar species or a species richness creep problem (i.e. that surveys show increasing richness over time as implementation of new protocols may include rarer and incidentally caught species as interest in all components of the ecosystem grows in the research community). This represents a broadening of the assemblage such that species which may have been confined to a smaller subset of sampling stations in the past are now found at more stations and the greatest rate of change occurred from about 1984–2004. The random effect for species (Model 4) captures some of the effect of this change in assemblage which could be almost 20% of the variance in the occupancy abundance relationship (Table 1).

## Discussion

Extinction debt theory (EDT) predicts that ecosystems experiencing permanent habitat destruction will accrue a debt on local species extinction (extirpation) where the best competitors will be the first species to disappear from the system [2,3]. It is a debt in the sense that it does not occur in the generation first experiencing the destruction but is paid slowly over multiple generations through declining fitness. The best colonizers will be less susceptible to habitat destruction because their increased dispersal ability allows them to find suitable habitats in a fragmented landscape even when total suitable habitat is decreasing. There is thus a colonizer credit where the destructive force gives colonizer species a relative competitive advantage. In fact, this credit can lead to longer term increases in community diversity: a corollary of EDT that may not be well appreciated [29]. In aquatic systems, one could expect EDT to be manifested in a decline in occupancy and abundance of competitor species at a faster rate than colonizer species. In marine systems this process is unlikely to lead to extirpation or extinction; instead, it more likely to be observed with long term changes in the occupancy of colonizers and competitors. Our analysis of the Georges Bank fish and invertebrate community shows a change in occupancy consistent with a moderated extinction debt where abundance standardized occupancy of competitor species decreased over time while it increased for colonizer species.

Donovan and Flather [30] showed that when habitats are fragmented and habitat quality is reduced there is a decreased occupancy of the fragmentation sensitive species (i.e., the equivalent of competitor species defined in this study). They speculated that the net result of local occupancy and a decrease in breeding success could lead to large scale population declines. Occupancy reduction of terrestrial animals in response to habitat destruction or fragmentation is common [23,31]. It is not unreasonable that the ecological occupancy responses in relation to habitat fragmentation may operate also in marine systems similar to bird populations which show habitat dependence in forested landscapes. The habitat dependence, however, may be more difficult to uncover in marine systems because of the technical challenges in identifying and classifying marine habitats, difficulties measuring changes in habitat fragmentation and problems identifying causes of the change in habitat quality and fragmentation. Additionally, most marine animals have large ontogenetically changing ecological roles and dependencies which can be manifested in very different habitat preferences as individuals age [32]. The fact that many marine species have highly dispersive larval stages and more sedentary adult stages means that extinction debt in a marine system would have terrestrial analogs as plants when young and birds when old. There are certainly added challenges to application of EDT in marine systems.

Georges Bank has been highly targeted with benthic trawling gear since industrial fishing began in earnest in the 1950s [6]. Benthic trawling gear is known to be destructive to habitats especially those that have long recovery times [12,33–35]. Benthic trawling (scallop dredging and otter trawling) is the major human induced habitat modifier that has occurred on Georges Bank [14] and if habitat deterioration and fragmentation has occurred on Georges Bank then it is reasonable to assume benthic trawling is an important driver of habitat change. Exacerbating any habitat destruction or loss; however, could be a change in suitability of the persistent Georges Bank fish and invertebrate community by warming temperatures [16] and of course direct exploitation by commercial fishing activities.

The decline in occupancy of competitor species on Georges Bank from the early 1960s until 2008 was undoubtedly affected by fishing activities which decreased their abundance. This alone would lead to a decline in occupancy of any species with a positive occupancy-abundance relationship [17,36]. This decline could have also depleted some sub-populations changing their relative contribution to overall abundance and consequently how the population uses space as a whole. The strength of the trends in the occupancy-abundance relationship have recently been confirmed and recreated through simulations [17] that assumed species statistical spatial distributions derived from natural communities and a simulated survey [37]. However, these studies showed that abundance was not the only factor explaining the occupancy decline and the nature of occupancy abundance relationship itself changed over time. Even if abundances were the same in 2008 as in 1963, less space was used to support that abundance [17]. Furthermore, refining this phenomenon showed that it was true for the whole community as well as within the competitor and mixed groups, but was the opposite for colonizer species. This decline in overall species occupancy, especially for competitor species, while colonizer species occupancy increased, is consistently explained by a decrease in habitat availability and/or suitability [14,38] and is broadly captured in an ideal sense by EDT.

George’s Bank has several closed areas to fishing established in 1994 [39]. These areas could have acted as refuges for competitor species in particular which may buffer a decline in competitor occupancy. The closed areas probably would have little effect on the transient effect of increased colonizer occupancy. The closed areas present an interesting possibility for future work in the sense that they create an experiment that could be subject to a relatively rigorous before-after press perturbation design. This experiment could possibly reveal impacts of the closed areas on the community from a perspective of EDT, i.e. the mix of community characteristics along the competitor-colonizer continuum.

The contraction of abundance into a smaller area for competitor species means that these populations are more vulnerable to fishing activities than they were previously [17,38]. Spatial contraction of a population is known to be an important factor to consider in the sustainable management of commercially exploited species especially if the fishery catch per unit effort is interpreted as an indicator of population abundance [40,41]. The decoupling of population size with commercial catch per unit of effort that can occur with population spatial contraction can lead to over-exploitation of a harvested population. It must be noted though that much of this increased biomass since 1994 is likely to be in closed areas [39] and therefore there may be less concern about overexploitation of the community since an important portion of that biomass remains unavailable to fishing.

Sea surface temperatures (SST) have been anomalously warm on Georges Bank since 1970 compared to most of the previous 70 years and those anomalies were greater in more recent years suggesting a possible regime in SST [42]. These increasing temperatures could have decreased the suitability of Georges Bank for colder water adapted species particularly demersal competitor species which may require greater environmental stability [16]. This suggests that temperature increases could also contribute to observed declines in the Georges Bank fish and invertebrate community abundance but not so much occupancy, at least not as a direct impact; but occupancy changes could be mediated through foodweb alterations that new species may have on the assemblage.

EDT is an approach first described in a modelled system based on mixes of species with perfect tradeoffs between competitive and colonizing abilities. EDT provides a useful basis for interpreting trends in occupancy-abundance relationships and species richness. However, the approach is limited in its current form for several reasons. Most importantly it is an equilibrium theory which assumes habitat destruction is permanent in a closed system without immigration or emigration. The cobble habitats, which are characteristic of Georges Bank fishing areas, have a regenerative capacity so their destruction is not usually permanent even if not immediately regenerated. Rescue effects from adjacent areas may also be possible, even if the areas adjacent to Georges Bank (Gulf of Maine, Scotian Shelf, Southern New England Banks) have experienced large declines in fish community abundance but these assemblage species still exist in these other areas. The second major issue with the tenets of EDT in a practical context is that most species have mixed strategies with both competitive and colonizing characteristics and cannot be so generally categorized. This is why our mixed group here contains so many species while start and end points of the continuum have fewer species.

Application of EDT in a marine system led to a useful analysis of community occupancy over time and provided a broad causal mechanism for its change (habitat fragmentation and destruction). The habitat forcing mechanism may be exacerbated by climate change and could also affected by direct impacts of fishing. There are conservation implications for this work in that competitor type species presently may be more vulnerable to fishing activities than they were in the 1960s although protected areas still shelter significant important portions of this biomass. Our combined theoretical and empirical approach gives insights into possible causes of trends observed for the Georges Bank fish community when viewed from the perspective of competitors and colonizer functional groups.

## Supporting Information

S1 Appendix list of species caught by the survey and analysed here. The small data set (39 species) are listed first and have a competitor-colonizer group classification while the large data set (164 species) includes the small set as well as other species which were considered reliably caught in a presence absence sense but perhaps not in terms of quantitative relative abundance and they were not assigned to a competitor-colonizer group.
(DOCX)
