# Supplementary material for: Extinction Debt and Colonizer Credit on a Habitat Perturbed Fishing Bank
Source: PLoS One. 2016 Nov 28;11(11):e0166409. doi: 10.1371/journal.pone.0166409 (PMC5125594; doi:10.1371/journal.pone.0166409)
Supplement: S1 Appendix — The small data set (39 species) are listed first and have a competitor-colonizer group classification while the large data set (164 species) includes the small set as well as other species which were considered reliably caught in a presence absence sense but perhaps not in terms of quantitative relative abundance and they were not assigned to a competitor-colonizer group. (DOCX) [file pone.0166409.s001.docx]

| **NMFS species code** | **Common name** | **Scientific Name** | **CC group** |
| --- | --- | --- | --- |
| 155 | Acadian redfish | *Sebastes fasciatus* | Competitor |
| 301 | American lobster | *Homarus americanus* | Colonizer |
| 102 | American plaice | *Hippoglossoides platessoides* | Mixed |
| 73 | Atlantic cod | *Gadus morhua* | Mixed |
| 32 | Atlantic herring | *Clupea harengus* | Colonizer |
| 121 | Atlantic mackerel | *Scomber scombrus* | Colonizer |
| 313 | Atlantic rock crab | *Cancer irroratus* | Colonizer |
| 156 | blackbelly rosefish | *Helicolenus dactylopterus* | Competitor |
| 131 | butterfish | *Peprilus triacanthus* | Colonizer |
| 176 | cunner | *Tautogolabrus adspersus* | Mixed |
| 84 | cusk | *Brosme brosme* | Competitor |
| 83 | fourbeard rockling | *Enchelyopus cimbrius* | Mixed |
| 104 | fourspot flounder | *Paralichthys oblongus* | Mixed |
| 197 | goosefish | *Lophius americanus* | Competitor |
| 109 | Gulf Stream flounder | *Citharichthys arctifrons* | Mixed |
| 74 | haddock | *Melanogrammus aeglefinus* | Mixed |
| 322 | lady crab | *Ovalipes ocellatus* | Mixed |
| 26 | little skate | *Leucoraja erinacea* | Competitor |
| 79 | longfin hake | *Urophycis chesteri* | Mixed |
| 503 | longfin squid | *Doryteuthis pealeii* | Colonizer |
| 163 | longhorn sculpin | *Myoxocephalus octodecemspinosus* | Mixed |
| 161 | moustache sculpin | *Triglops murrayi* | Mixed |
| 181 | northern sand lance | *Ammodytes dubius* | Colonizer |
| 502 | northern shortfin squid | *Illex illecebrosus* | Colonizer |
| 193 | ocean pout | *Macrozoarces americanus* | Mixed |
| 75 | pollock | *Pollachius virens* | Mixed |
| 77 | red hake | *Urophycis chuss* | Mixed |
| 164 | sea raven | *Hemitripterus americanus* | Mixed |
| 401 | sea scallop | *Placopecten magellanicus* | Colonizer |
| 72 | silver hake | *Merluccius bilinearis* | Mixed |
| 27 | smooth skate | *Malacoraja senta* | Competitor |
| 15 | spiny dogfish | *Squalus acanthias* | Competitor |
| 28 | thorny skate | *Amblyraja radiata* | Competitor |
| 76 | white hake | *Urophycis tenuis* | Mixed |
| 108 | windowpane | *Scophthalmus aquosus* | Mixed |
| 106 | winter flounder | *Pseudopleuronectes americanus* | Mixed |
| 23 | winter skate | *Leucoraja ocellata* | Competitor |
| 107 | witch flounder | *Glyptocephalus cynoglossus* | Mixed |
| 105 | yellowtail flounder | *Limanda ferruginea* | Mixed |
| 297 | aesop shrimp | *Pandalus montagui* | - |
| 33 | alewife | *Alosa pseudoharengus* | - |
| 165 | alligatorfish | *Aspidophoroides monopterygius* | - |
| 384 | American eel | *Anguilla rostrata* | - |
| 35 | American shad | *Alosa sapidissima* | - |
| 173 | armored searobin | *Peristedion miniatum* | - |
| 46 | Atlantic argentine | *Argentina silus* | - |
| 199 | Atlantic batfish | *Dibranchus atlanticus* | - |
| 123 | Atlantic bonito | *Sarda sarda* | - |
| 1 | Atlantic hagfish | *Myxine glutinosa* | - |
| 101 | Atlantic halibut | *Hippoglossus hippoglossus* | - |
| 36 | Atlantic menhaden | *Brevoortia tyrannus* | - |
| 132 | Atlantic moonfish | *Selene setapinnis* | - |
| 205 | Atlantic saury | *Scomberesox saurus* | - |
| 170 | Atlantic seasnail | *Liparis atlanticus* | - |
| 113 | Atlantic silverside | *Menidia menidia* | - |
| 262 | Atlantic soft pout | *Melanostigma atlanticum* | - |
| 403 | Atlantic surfclam | *Spisula solidissima* | - |
| 21 | Atlantic torpedo | *Torpedo nobiliana* | - |
| 192 | Atlantic wolffish | *Anarhichas lupus* | - |
| 204 | banded rudderfish | *Seriola zonata* | - |
| 22 | barndoor skate | *Dipturus laevis* | - |
| 517 | bathyal swimming crab | *Bathynectes longispina* | - |
| 43 | bay anchovy | *Anchoa mitchilli* | - |
| 263 | beardfish | *Polymixia lowei* | - |
| 134 | bigeye | *Priacanthus arenatus* | - |
| 876 | bigeye cigarfish | *Cubiceps pauciradiatus* | - |
| 209 | bigeye scad | *Selar crumenophthalmus* | - |
| 141 | black sea bass | *Centropristis striata* | - |
| 114 | blackmouth bass | *Synagrops bellus* | - |
| 81 | blue hake | *Antimora rostrata* | - |
| 129 | blue runner | *Caranx crysos* | - |
| 34 | blueback herring | *Alosa aestivalis* | - |
| 135 | bluefish | *Pomatomus saltatrix* | - |
| 120 | bluespotted cornetfish | *Fistularia tabacaria* | - |
| 228 | boa dragonfish | *Stomias boa* | - |
| 296 | bristled longbeak | *Dichelopandalus leptocerus* | - |
| 112 | buckler dory | *Zenopsis conchifera* | - |
| 616 | bulleye | *Cookeolus japonicus* | - |
| 454 | Carolina hake | *Urophycis earlli* | - |
| 124 | chub mackerel | *Scomber japonicus* | - |
| 24 | clearnose skate | *Raja eglanteria* | - |
| 321 | coarsehand lady crab | *Ovalipes stephensoni* | - |
| 511 | common octopus | *Octopus vulgaris* | - |
| 63 | conger eel | *Conger oceanicus* | - |
| 183 | daubed shanny | *Lumpenus maculatus* | - |
| 158 | deepbody boarfish | *Antigonia capros* | - |
| 110 | deepwater flounder | *Monolene sessilicauda* | - |
| 194 | fawn cusk-eel | *Lepophidium profundorum* | - |
| 175 | flying gurnard | *Dactylopterus volitans* | - |
| 295 | friendly blade shrimp | *Spirontocaris liljeborgii* | - |
| 556 | glasseye snapper | *Priacanthus cruentatus* | - |
| 202 | gray triggerfish | *Balistes capriscus* | - |
| 99 | Greenland halibut | *Reinhardtius hippoglossoides* | - |
| 166 | grubby | *Myoxocephalus aenaeus* | - |
| 54 | horned lanternfish | *Ceratoscopelus maderensis* | - |
| 340 | Iceland scallop | *Chlamys islandica* | - |
| 312 | jonah crab | *Cancer borealis* | - |
| 206 | longnose batfish | *Ogcocephalus corniger* | - |
| 133 | lookdown | *Selene vomer* | - |
| 168 | lumpfish | *Cyclopterus lumpus* | - |
| 208 | mackerel scad | *Decapterus macarellus* | - |
| 65 | margined snake eel | *Ophichthus cruentifer* | - |
| 91 | marlin-spike | *Nezumia bairdi* | - |
| 869 | mooneye cusk-eel | *Ophidion selenops* | - |
| 116 | northern pipefish | *Syngnathus fuscus* | - |
| 196 | northern puffer | *Sphoeroides maculatus* | - |
| 171 | northern searobin | *Prionotus carolinus* | - |
| 694 | northern sennet | *Sphyraena borealis* | - |
| 306 | northern shrimp | *Pandalus borealis* | - |
| 324 | northern stone crab | *Lithodes maja* | - |
| 299 | Norwegian shrimp | *Pontophilus norvegicus* | - |
| 69 | offshore hake | *Merluccius albidus* | - |
| 185 | oyster toadfish | *Opsanus tau* | - |
| 292 | pink glass shrimp | *Pasiphaea multidentata* | - |
| 201 | planehead filefish | *Monacanthus hispidus* | - |
| 294 | polar lebbeid | *Lebbeus polaris* | - |
| 285 | punctate blade shrimp | *Spirontocaris phippsii* | - |
| 184 | radiated shanny | *Ulvaria subbifurcata* | - |
| 45 | rainbow smelt | *Osmerus mordax* | - |
| 489 | red cornetfish | *Fistularia petimba* | - |
| 310 | red deepsea crab | *Geryon quinquedens* | - |
| 862 | redeye gaper | *Chaunax stigmaeus* | - |
| 180 | rock gunnel | *Pholis gunnellus* | - |
| 25 | rosette skate | *Leucoraja garmani* | - |
| 212 | rough scad | *Trachurus lathami* | - |
| 31 | round herring | *Etrumeus teres* | - |
| 211 | round scad | *Decapterus punctatus* | - |
| 143 | scup | *Stenotomus chrysops* | - |
| 287 | sevenspine bay shrimp | *Crangon septemspinosa* | - |
| 631 | sheepshead | *Archosargus probatocephalus* | - |
| 508 | shield bobtail | *Stoloteuthis leucoptera* | - |
| 557 | short bigeye | *Pristigenys alta* | - |
| 162 | shorthorn sculpin | *Myoxocephalus scorpius* | - |
| 232 | shortnose greeneye | *Chlorophthalmus agassizi* | - |
| 865 | silver anchovy | *Engraulis eurystole* | - |
| 213 | silver rag | *Ariomma bondi* | - |
| 66 | silverstripe halfbeak | *Hyporhamphus unifasciatus* | - |
| 67 | slender snipe eel | *Nemichthys scolopaceus* | - |
| 117 | smallmouth flounder | *Etropus microstomus* | - |
| 13 | smooth dogfish | *Mustelus canis* | - |
| 182 | snakeblenny | *Lumpenus lumpretaeformis* | - |
| 325 | snow crab | *Chionoecetes opilio* | - |
| 518 | speckled swimming crab | *Arenaeus cribrarius* | - |
| 293 | spiny lebbeid | *Lebbeus groenlandicus* | - |
| 512 | spoonarm octopus | *Bathypolypus arcticus* | - |
| 78 | spotted hake | *Urophycis regia* | - |
| 798 | spottedfin tonguefish | *Symphurus diomedianus* | - |
| 856 | striated argentine | *Argentina striata* | - |
| 44 | striped anchovy | *Anchoa hepsetus* | - |
| 139 | striped bass | *Morone saxatilis* | - |
| 103 | summer flounder | *Paralichthys dentatus* | - |
| 115 | threespine stickleback | *Gasterosteus aculeatus* | - |
| 596 | vermilion snapper | *Rhomboplites aurorubens* | - |
| 240 | viperfish | *Chauliodus sloani* | - |
| 229 | weitzmans pearlsides | *Maurolicus weitzmani* | - |
| 280 | western softhead grenadier | *Malacocephalus occidentalis* | - |
| 246 | white barracudina | *Arctozenus rissoi* | - |
| 190 | wolf eelpout | *Lycenchelys verrilli* | - |
| 191 | wrymouth | *Cryptacanthodes maculatus* | - |
| 237 | luciérnaga musculosa | *Polymetme thaeocoryla* | - |
| 245 | sharpchin barracudina | *Paralepis coregonoides* | - |
| 251 | slope hatchetfish | *Polyipnus clarus* | - |
| 298 | *Atlantopandalus propinqvus* | *Pandalus propinquus* | - |
| 867 | shortbeard codling | *Laemonema barbatulum* | - |
